# Cullings from Contemporaries

**Published:** 1888-03

**Authors:** 

**Affiliations:** Austin, Texas


### CULLINGS FROM CONTEMPORARIES.

*Edited by T. J. Bennett, M. D., Austin Texas.*

RESTORED TO SIGHT AFTER SIXTY YEARS.—Dr. Dav. McKeowan, in *The Lancet*, for January last, reports the case of a fiddler who had been blind over 60 years, caused by small pox when a child. Iridectomy was performed, and the result was restoration to fair sight. Strange he had gone thus long. His color education was said to be very rapid. He learned to distinguish the coins first.

BENZOATE OF SODA IN URÆMIA.—According to Dr. A. S. Partzersky, of Moscow, this drug acts almost as a specific in uræmic poisoning. Ten cases were treated, seven of which suffered from parenchymatous and three from interstitial nephritis. The dose was 1 to 2 drachms daily. Nine of the patients recovered, one died. Headache, dilation of pupils, albuminuria and even the convulsive phenomena disappear. Its use is recommended to commence with the first symptoms.

NATIONAL CONTROL OF MARITIME QUARANTINE.—The College of Physicians and Surgeons, Philadelphia, has set in motion an effort to place the maritime quarantine and all sanitation under the control of the general government. A committee has been appointed consisting of Drs. J. C. Wilson, E. O. Shakespeare and R. A. Cleeman, to induce the present congress to adopt such a law. An address has been issued asking the cooperation of all county, district and State organizations.

REMOVE THE DUTY ON FOREIGN MEDICAL SUPPLIES.—The Georgia Medical Society has moved in the right direction, At its January meeting the following resolution was unanimously adopted .

*Resolved,* That the corresponding secretary enter into correspondence with other State and local medical societies, with the view to induce them to act in concert with us in an effort to influence Congress to remove the *Import Duty* from all medical and surgical supplies, instruments and appliances.

Sterility in the Male.—Dr. Kehrer, of Heidelberg, has arrived at the conclusion, after a series of well conducted experiments, "that in at least a third of the cases of sterile marriages the husband was the party at fault, and gonorrhœa was the cause of the barrenness."

---

Scheele's Test for Abdominal Aneurism.—Make firm simultaneous compression of both femoral arteries for a few seconds. If there be aneurism the patient will immediately complain of pain in and around the tumor. Otherwise he will experience no special discomfort.

---

Comparative Statistics of the Treatment of Diphtheria.—Dr. Lunin, of St. Petersburg, gives the following trial of various remedies in the treatment of diphtheria in the Oldenburg hospital: Cases, 296 ; percentage of deaths, 55.

FIBRINOUS FORM—DEATHS:

| | | |
|---|---|---|
| By turpentine | 8.30 | per ct |
| resorcine | 20.00 | " |
| sublimate | 30.20 | " |
| chinoline | 31.60 | " |
| fer. perchlor | 32.60 | " |
| bromine | 46.7 | " |

SEPTIC PHLEGMONOUS FORM—DEATHS :

| | | |
|---|---|---|
| By turpentine | 81.00 | per ct |
| resorcine | 89.5 | " |
| sublimate | 92.5 | " |
| chinoline | 100 | " |
| ferri perchlor | 76.5 | " |
| bromine | 86.9 | " |

GENERAL RESULT—DEATHS :

| | | |
|---|---|---|
| By turpentine | 43.4 | " |
| resorcine | 65 | " |
| sublimate | 45 | " |
| chinoline | 53 | " |
| ferri perchlor | 56 | " |
| bromine | 69.7 | " |

Cocaine in Nasal Polypus and Hemorrhoids.—In *Albany Medical Annals* for February it is stated that 10 to 15 drops of a six per cent. solution injected into a polypus or a hemorrhoid will cause its death by contraction of the blood vessels that supply it. Then why should cocaine not cure uterine fibroids hypertrophiəd tonsils and goitres in the same way.

---

How To Make Splints.—Brush over thick woolen cloth a strong alcoholic solution of shellac. If it is desired to have the splint very stiff place several pieces of the cloth together and pass a hot iron over them. They can be softened by hot water and moulded and cut in any desired shape.

---

Vessication For Vomiting Of Pregnancy.—A writer in the *Lancet* says that vessication over the fourth and fifth dorsal vertebræ never fails to put an end to reflex vomiting for the whole remaining period of gestation, no matter at what stage it is effected.

---

Jaborandi In Erysipelas.—Prof. Waugh prescribes twenty drops of the fluid extract every two hours until perspiration sets in. If the erysipelas shows a tendency to recur, the use of the drug is resumed. *Albany Medical Annals.*

---

Gravity As An Expectorant.—The *Polyclinic* contains the suggestions that inverting a patient and causing him to cough while in this position will unload the bronchial tubes of mucous secretion and avert impending death.

---

Ichthyol In Skin Diseases.—Ichthyol is a clear yellow-brown oil obtained by distilling bituminous matter which contains the fossilized remains of fishes. It is fast coming to the front as a remedy in all kinds of skin diseases. The Philadelphia County Medical Society had it up last month as an antiseptic in surgery. It is destined to the same wide range as iodoform, especially for odor.

---

Tic-dolereux Cured by Neurectomy of Three Branches of the Trifacial.—In the *International Journal of Surgery and Antiseptics*, Dr. A. C. Bernays, of St. Louis, reports a unique case of tic-doulereux of 26 years duration entirely relieved by resecting, at one sitting, three divisions of the trigeminus. The patient was 69 years of age. The microscope revealed nothing abnormal in the structure of the nerves.

---

How to Use Electricity in Extra Uterine Pregnancy.—Dr A. D. Rockwell, of New York, in the *International Journal of Surgery and Antiseptics*, details the directions for employing electricity for the destruction of the fœtus in the tubal pregnancies, which boiled down, are as follows: First make the diagnosis. Then place the positive electrode firmly upon the abdomcn and directly over the distended tube. The negative electrode, consisting of a metal ball about the size of a large marble, securely fastened to an insulated stem, is introduced into the vagina, and guided to the point where the enlargement is most distinctly felt. The interrupted galvanic current is prefered;strength between fifteen and twenty millometres.

---

### ROETHELN.

---

LETTER TO EX-PRESIDENT A. P. BROWN, OF FORT WORTH, FROM DR. T. C. OSBORN.

*Dr. A. P. Brown, Fort Worth.*

Dear Sir:—For a copy of the *Weekly Medical Review* of November 12, containing an article on "Roetheln," by Dr, Henry Davis, of Tuam, sent me at your request from the office in St. Louis, I beg to return you my sincere thanks.

The subject is very ably written, and suggestive of great familiarity with the disease, and inspires confidence in the reader in the honesty and ability of Dr. Davis, an observer of no mean pretensions.

The first paragraph of the paper contains nearly all that is positively known of the disease, and any difference that might be suggested by a critical reader would consist mainly in seeing the disease from a different standpoint, without changing in the least the most important features of roetheln as an affection having a definite anatomy. Even the wide difference of opinion amongst writers as to its hybridity can be easily reconciled by the admission that it is conclusively "the missing link" between the two diseases, rubeola and scarlatina, which it so faithfully represents in every way except in its infectious nature. And as hybrids do not, as a rule, propagate their own species; and as roetheln is, according to my observation, not at all infectious in its nature, my own opinion leans strongly, if not conclusively, to the belief in the hybridity of the disease. But admitting the mulish appearance of the complaint, I still cannot see why its autonomy should be doubted.

Regarding the disease as dysthetic, recurrent and non-contagious, I am quite prepared to agree with Dr. Davis that roetheln rather increases than diminishes the liability of its invasion. But whether this disposition will ever be confessed as to creating an increased liability to other diseases is, in my estimation open to grave doubt. It is, nevertheless, true that the dyscrasia upon which the disease depends does not for many days leave the system, and to this special diathesis, doubtless, the doctor had reference in making the suggestion. It is well known, that local epidemics may, and that general epidemics, of every kind, invariably do impose upon communities decided peculiarities, and sometimes lingering diatheses differing materially from that which previously existed in the organism.

This is assuredly seen after the periodical returns of the great malarial fever epidemics which occur with something like regularity every fifteen to twenty years apart. That, for instance, of 1840, created a diathesis of so decided a nature as to influence the profession everywhere against venesection, a practice which had existed from time immemorial, and like many other useful agents was, no doubt, often terribly misused in the hands of its sanguine partisans; and that of 1867, which again changed diathesis, renewing in a quiet way an apparent necessity for discussing bloodletting as a measure of relief in inflammatory conditions. These instances are merely mentioned as illustrative o- the influence exerted upon individuals and communities by epideml ics of a non-contagious disease, whether they occur from malariaf toxemia, or from areas of low barometer with all its other usual concomitants, such as an excess or want of atmospheric electricity, temperature and humidity, And there can be but little hesitation in ascribing these conditions as really the true operative causes of the disease we are describing.

Roetheln, like diphtheria in the South, dengue, rheumatism, asthma and neuralgia, is mainly dependent upon meteorological conditions, and as areas of low barometer are very often limited in circumference, the reason is plain why these affections are frequently observed in certain places and not in other, although it may be adjacent, neighborhoods.

According to the observations I have made in several epidemics through which my labors have passed, or rather, from my standpoint, I am forced to differ with Dr. Davis in the assertion that roetheln more frequently resembles scarlatina than it does measles.

There is in my estimation but a single feature, which, although very constant in all the cases I have witnessed, is, nevertheless, merely a shadowed resemblance to the anginose condition of scarlatina, and very rarely, if ever, invades the palate and posterior nores nor is there the same amount of intensity of engorgement of the tonsils, and suffusion of the mucous membrane of the fauces, which is observed in the simplest forms of scarlet fever. Whilst, on the other hand, there is never a case seen where the eruption could for a moment be mistaken for that which constitutes the characteristic blush of scarlatina.

But the differentiation between roetheln and rubeola is much more difficult, owing to the similarity of the maculated appearance of the skin, the sneezing, and the cough so constantly seen in measles.

Indeed the color and general outlines of the eruption constantly force the observer to ask the patient if he has ever had measles, and then, perhaps, for the first time, if answered in the affirmative, the eruption is found not to be crescentic, but roundish, oval, or oblong, and disposed to be gregarious. There is the slightest imaginable difference in the color of the muscles between roetheln and rubeola.

But, after all, I am only speaking of the disease as I have seen it in several epidemics in Alabama and in Texas, and do not mean to call in question the observations of other observers, knowing, as I do, that the disease very frequently presents protean features almost defying rational discrimination.

The most important consideration is that roetheln is too often confounded with other diseases, rather than on its own account. In April, 1886, two children in one family were attacked with what was said to be measles. There was no more than a day or two difference in the dates of the inception; the youngest, a *suckling*, aged five month, a girl, died after one week of illness; the other, a boy, aged two years and a half, died one week later. There was no other case of measles in the city, nor had the children been exposed to the infection. These death occurred in the same house, and although there were others in the family, and in the neighborhood, openly exposed to the contagion from these cases, yet there was no spread of the disease. In the beginning of the ensuing month, May, a school girl, aged eight years, in another part of the city, was ill with the same disease, but soon recovered without treatment, and no other cases were then, or afterward, infected in the classes of the school.

A day or two after the attack, her baby brother, a *suvkling*, eight month old, was taken similarly, and after one week's illness died of congestion of the lungs, having the eruption of measles, and the sore throat of scarlet fever. The day before the death of this child, I saw a lady, mother of two children, who asked me to prescribe for her throat, and when I visited her the entire surface of her face, neck, body and extremities was covered with an eruption so apparently like measles that I incautiously called it so, but she at once reminded me that she had had measles several years before. Upon this I inspected the eruption more closely and found it wanting in crescentic shapes, and very much bunched on the extremities; the fauces and tonsils were red and tumid, but without ulceration, or the punctated appearance peculiar to scarlatina. The tongue was clean, with very few papillæ. There was barely one degree of fever, and the pulse was normal. There had been no sneezing, and no cough.

Seeing now plainly that it was a case of roetheln, I asked, if she had been with cases like it? She had not, but heard there was a similar case in the neighboring house, to which I made my way, and asked permission to see the child, and this proved to be the dying child mentioned above.

On close inspection I found the body and extremities covered with an eruption like measles, but growing rapidly paler as the life of the child ebbed away, and with the sore throat, but no cough nor sneezing. Evidently it was another case of roetheln, and died that night from congestion of the lungs, brought on by undue exposure to a draft of night airs. No other cases occurred in this neighborhood after these. The children of my lady patient did not have the disease, although one of them was *suckling*. Now, the cases so far reported were in neighborhoods similar in appearance and upon the same low level, but there was not the least intercourse of a social nature between the families affected. Afterwards cases of the diseases in other parts of the city and the surrounding country occurred, making up an epidemic, but in none of them could it be said that a positive infectiousness exists. In June a bright little girl in the higher location of the city was attacked, and the attending physician treated the case as scarlatina, and it died after four days illness. In the next September another case occurred in the flat, not far from my lady patient, of a *suckling* ten months old and, after lingering three weeks, died apparently from extreme exhaustion. I saw this case in consultation, and insisted upon its being rotheln, and strongly urged a discontinuance of the application of ice water to the head, asserting that local application of cold would certainly induce internal congestion, and the temperature (105 °) would not necessarily destroy life, if the powers of nature were properly conserved in the mean time. But the attending physician did not agree with me, and next day called in consultation the same physician who had treated what he called an exactly similar case in June, or the case called scarlatina noticed above. He advised the ice water, and the child died. In one cf the cases, the attending physician declared to me his firm belief, that the disease was scarlatina anginosa. Upon which I remarked, "you must not have seen cases of scarlatina anginosa very often then."

Here then, in the city of Cleburne, were five deaths from what was undoubtedly the disease known as roetheln, and which were confounded with other diseases, namely, rubeola, and scarlatina. And I underscored the sucklings, because Hebra; Wilson, Hillier, and others claim immunity from the disease in this class of subjects.

In defense of my theory that epidemics of roetheln—there is I believe, never an instance of the sporadic, isolated form of the disease on record—it is necessary to mention in an intercalary manner the condition of the prevailing weather ststistics of Cleburne during the season from March to October, 1886.

We were in the midst of a long drought; the temperature did not at any time get above 98°. Fahrenheit; the humidity was unusually low; thunder storms were remarkably infrequent; end the barometer was steadily below its normal readings; which, all taken together, was amply sufficient to induee dysthetic skin diseases, mainly of the non-contagious order, and, I will add, that in this county, and, indeed, several other counties adjacent to this, there was a remarkable number of eruptive diseases prevailing at the time of the existence of the epidemic of roetheln. Of these eruptions eczema largely overshadowed all others in the order of frequency, and there were really but few persons upon whom this, or some other skin trouble did not exist at some time during the season mentioned.

Now, then, whilst I am quite willing to admit exceptions to the rule, I will, nevertheless, assert in a positive manner that such seasons as that given will in any section of the world, produce almost identically the same, or similar epidemics; as those witnessed in this place between the months of March and October 1886.

From this declaration of my opinion, you may justly infer that I am not theatrically posing in the attitude of a bacteriologist. On the contrary, I am far from believing that we are advancing at any great rate in the elucidation of diseases by thrusting a micrococcus before the world as the specific *cause*, whilst the fact remains still undisputed that the bacteria may just as likely be the *result* of the disease.

The simplest cases are the typical cases of roetheln, and any departure or increase in violence of the disease, either leaning toward rubeola or scarlatina, is mainly due to the depth of the dyscrasia in the blood, due to the peculiar constitution of the atmosphere in which the victims have for sometime lived, and to prior dveiations of health induced by other etiological influences. It is of the typical cases only that a correct idea of the disease can be rationally formed.

In a typical case we find that a husky feeling of the throat, with some little trouble in deglutition are the earliest symtoms noticed, which, in the course of two or three days, is followed by a light degree of fever, and, simultaneously, an eruptions appears over the face, neck, body, and extremities, so nearly resembling measles that a closer inspection is required to discriminate between them. The eruption is never crescentic as in measles, and is prone to coalesce, or bunch, especially on the extremities, but the color and other features of the maculæ are almost identical with rubeola. The pulse seldom varies much from a healthy standard, and in this we have a wide differece between that of either measles, or scarlet fever.

The appearance of the eruption and the fever occur usually about the second or third day of the fever, and both disappear at the end of two or three days, when a branny desquamation sets in more or less abundantly on the body, but greatest on the fingers and toes; and at this time there is scarcely any of the soreness of the throat left to annoy the patient. But there is one pathognomonic feature which is eminently distinctive of roetheln, one; indeed, so constant in its occurrence that when observed there can be no longer a footing for dcubt as to its true character, and that is an enlargement of the small glands just at the edge of the hair of the head on the postero-lateral sides of the neck. This feature has not been absent in any case coming under my own, or the observation of any other observer to whom cases of the disease have occurred. Such glandular enlargements do not occur in either scarlatina or measles, but are distinctively characteristic of roetheln. Even in these departures in violence leaning toward either measles or scarlet fever, these swollen glands aae never absent, but are larger as the disease becomes most virulent in its nature, especially if the bearing is toward scarlatina.

The departures may be so violent as to threaten the life of the patient, and are mainly due to tendencies to internal congestion from imprudent management on the part of the patient, or the nurse, or to prior vice in the system, Death may occur from coma or convulsions, from congestion of the lungs, or from disorganization of the kidneys, but as there has been no necroscopy recorded, I am at quite at loss to say what are the footprints of the disease after death. Indeed, when death has occurred, there can be no question as to its being cuased by complication, and, therefore, in an autopsy we would naturally expect to find only such traces as are necessarily those of the complication. Without inter-current complications we would never expect to find a death from this simple disease on record. The clinical history is the most we can boast of in our day.

But, as I have asserted the non-contagiousness of the disease, it is but just that I should defend my position, especially as roethlen is, with few exceptions, declared to be infectious by a majority of the observers who have written upon the subject.

In the first place, the first cases in my experience with several epidemics have sprung into existence in each instence amongst people who had in no way been exposed to infection, and it was more to the number of the cases occurring under the same meteorological conditions that the idea to others has given rise to the suspicion of contagiousness, forgetful of the old axiom that, "that which has been, will, under the same circumstances, be so again."

Too often to admit of doubt, I have seen only one out of a large family of children in the same house go through the disease, none of the others subsequently having it.

On the other hand, I have often witnessed the entire family stricken almost at the same moment, and in which there had been no communication with perhaps, distant cases in other families. And, lastly, I have done my best to prove its infectiousness by exposing my own children to it, without ever an instance confirmatory of the the theory of contagion, nor did I have the disease myself.

Dr. Sholl, of Sumter county, Alabama, who is a close observer, states that he could not trace beyond the first two or three cases, and yet he contends bravely for the infectiousness of the disease in the epidemic of 1880. On the contary, I was at the same time but forty miles from him, and the midst of a similar epidemic, in Hale county, Alabama, where my opportunities were equal to his, but where I was forced to the conclusion of its non-contagiousness.

But, after all, he and I cordially agreed in the conclusion that the disease in many instances required all our exertion to prevent ugly complications which would, if allowed to enter as an intercurrent, result seriously to the life of the patient we had under treatment.

Ane now, my dear Doctor, hoping I have not jaded you by my prosaic composition, and that you will credit me with only the best intentions, I beg to subscribe myself with profound respect.

Yours, etc.,

T. C. OSBORN.

---

THE MEDICAL AND SURGICAL HISTORY OF THE WAR OF THE REBELLION.—The limited edition of this great work, issued in accordance with an act of Congress, is entirely exhausted. There is great demand for the work, and as it was stereotyped, and the plates are still in existence, another edition could be easily produced. In the *Therapeutic Gazette*, February 15, Dr. C. M. Fulton has a letter on the subject, in which he requests cooperation in an effort to secure action of Congress in the matter. We heartily concur in the opinion that another issue would meet with general favor and large sale, and join the Doctor in the hope that Congress may order it.

---

THE BRAZOS AND COLORADO MEDICAL ASSOCIATION has been organized, with Dr. Bat. Smith, of Wharton, President. This organization will send delegates to the State Convention at Galveston, April 24, 1888.

---

MICROBE AND MICROBE KILLERS.—There is a citizen of Austin by the name of L. E. Daniell, and he is engaged in the manufacture and sale of a proprietary article called Microbe Killer or Microbe Destroyer. The press, with that very prevalent disposition to pay questionable compliments to certain people, have daubed Mr. Daniell "Doctor," and the very close resemblance of his name (especially with the prefix) to that of the Editor of this Journal, has created the belief in the minds of some people that we are the party. Now this is most exasperating! We have been seriously asked about it! We know that those who know us, and those for whose opinion we have any or much regard, could not believe us capable of such a thing; yet, it is annoying to be so mixed up with such a subject. But what can we do?—This is to all to whom it may come, greeting,—and to inform them that we have no connection with any such business as Microbe Killers, and we do not wish to be counfounded with L. E. Daniell—the alleged "Doctor" who is selling the alleged Microbe Destroyer.

